# The role of income inequality and social policies on income-related health inequalities in Europe

**DOI:** 10.1186/s12939-015-0247-y

**Published:** 2015-10-31

**Authors:** Regina Jutz

**Affiliations:** GESIS - Leibniz Institute for the Social Sciences, Mannheim, Germany

**Keywords:** Health inequalities, Income, Income inequality, Social spending, Europe, EVS

## Abstract

**Introduction:**

The aim of the paper is to examine the role of income inequality and redistribution for income-related health inequalities in Europe. This paper contributes in two ways to the literature on macro determinants of socio-economic inequalities in health. First, it widens the distinctive focus of the research field on welfare state regimes to quantifiable measures such as social policy indicators. Second, looking at income differences completes studies on socio-economic health inequalities, which often analyse health inequalities based on educational differences.

**Methods:**

Using data from the European Values Study (2008/2009), 42 European countries are available for analysis. Country characteristics are derived from SWIID, Eurostat, and ILO and include indicators for income inequality, social policies, and economic performance. The data is analysed by using a two-step hierarchical estimation approach: At the first step—the individual level—the effect of household income on self-assessed health is extracted and introduced as an indicator measuring income-related health inequalities at the second step, the country-level.

**Results:**

Individual-level analyses reveal that income-related health inequalities exist all across Europe. Results from country-level analyses show that higher income inequality is significantly positively related to higher health inequalities while social policies do not show significant relations. Nevertheless, the results show the expected negative association between social policies and health inequalities. Economic performance also has a reducing influence on health inequalities. In all models, income inequality was the dominating explanatory effect for health inequalities.

**Conclusions:**

The analyses indicate that income inequality has more impact on health inequalities than social policies. On the contrary, social policies seemed to matter to all individuals regardless of socio-economic position since it is significantly positively linked to overall population health. Even though social policies are not significantly related to health inequalities, the power of public redistribution to impact health inequalities should not be downplayed. Social policies as a way of public redistribution are a possible instrument to reduce income inequalities which would in turn lead to a reduction in health inequalities.

## Introduction

Since Wilkinson [1] published *Unhealthy Societies: The Afflictions of Inequality*, many scholars have studied the effect of macro determinants on average population health. The number of studies on the relationship between the welfare state and average health, which recent reviews [2, 3] have examined, gives an impression of the significance of this area of research. Most findings indicate that an association exists between improved average population health—e.g., measured by life expectancy, infant mortality, self-reported health, or certain health symptoms—and egalitarian political traditions and welfare state generosity compared to conservative political traditions and low levels of welfare state spending [3]. However, regarding the variance of population health, a research gap persists.

The present study aims to narrow this research gap by providing insight into how socio-economic health inequalities are related to income inequality and social policies. Socio-economic inequalities in health mean that health outcomes vary according to socio-economic factors such as education, income, or occupation. The explanations about how these factors affect health are manifold, ranging from diverse psychosocial mechanisms [4] to material factors to differences in health-related behaviour [5, 6]. Psychosocial factors affect health directly, e.g. chronic stress affecting the immune system, and indirectly via health-damaging behaviours such as e.g. smoking [6]. The explanation which focuses on material factors is based on the lack of material resources (direct effect), which also indirectly affects health via psychosocial stress and health-related behaviour (e.g. malnutrition). Furthermore, health-related behaviour also contributes to health inequalities: E.g. lower social status groups show less attendance for preventive medical care [7].

The present study focuses on income-related health inequalities as income represents a household’s material condition and thus is a useful measure of socio-economic status [8]. Income creates material circumstances that affect health via the quality of housing, food, medical care, and opportunities for recreational and physical activities [5]. Looking at income differences complements studies on socio-economic health inequalities which often use education as indicator for socio-economic position [2]. Nevertheless, following Lahelma [9] who points out the interrelations of the key indicators of socio-economic position—education, occupational class, and income—I introduce education as control variable.

**Fig. 1:**
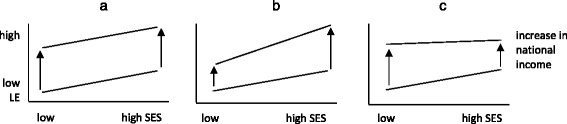
Different scenarios about how an increase in national income could affect health inequalities (arrows represent the size of health inequalities), using the example of life expectancy. Note: LE = Life expectancy; SES = Socio-economic status

It is important to not confuse determinants of health with determinants of *health inequalities* [10]. An increase in national income, meaning an increase in the standard of living, which would improve health, does not necessarily lead to decreasing health inequalities. If everyone benefits in the same way from a higher standard of living, the level of average health rises, but health inequalities could persist, as Fig. 1a shows. Link and Phelan [11] describe this as the fundamental cause approach: people with more socio-economic resources are able to maintain their health advantage over people with fewer resources. However, one also could imagine that higher socio-economic status (SES) groups benefit more from an increase in national income, e.g., via certain expensive medical innovations [12]. In this scenario, the number of people who could not afford medical treatment increases, and hence health inequalities also would rise (Fig. 1b). On the other hand, if lower SES groups benefit more than higher SES groups from an increase in living standards (e.g., secure housing becomes affordable for all), health inequalities are reduced (Fig. 1c).

These three depictions reveal that an automatism between increases in national income and health inequalities does not exist: the relationship depends on how the increases are distributed within a society. The distribution happens via two processes: first, by the distribution of personal earnings and second, by redistribution via social policies. Both processes indicate whether only a small elite or the broad public participates and benefits from economic wealth.

### Income inequality

Several reviews [13–15] address the influence of income inequality on population health. Even though the conclusions are mixed and only partly suggest a negative effect of income inequality on health, the authors acknowledged the relevance of income inequality for health *inequalities* [15]. Income inequality affects the average population health via two possible mechanisms. First, there is the neo-material perspective that suggests that the unequal distribution of income leads to underinvestment in human, physical, or cultural capital, as well as in the educational system and medical services [5, 16]. Privileged groups within a society are able to use privately managed services, and therefore, are no longer interested in maintaining public services. If the quality of public service provision is poor, households act rationally by opting out and investing in the private alternatives available, which further undermines the financing of public services [17]. Lower socio-economic status groups are more affected, since they are more likely to be dependent on public services and infrastructure, e.g., schools, transportation, and medical services. Higher income inequality would lead to a widening health gap between the people who have little income and who rely on public services, and those who do not.

The second mechanism is psychosocial: everyone in society is subject to social comparison; people look up and down the social ladder and evaluate their social status. Seeing that others are better off than oneself leads to increased stress levels, which eventually could damage mental and physical health [13]. As a consequence, all people of all income levels are affected by health inequalities—independent of absolute poverty. The observed income gradient in health often is interpreted as evidence of this mechanism [9, 18] since it describes how health worsens with every step down the social ladder.

Subramanian and Kawachi [19] studied the effect of income inequality on the subjective health of different population groups in 50 US states by applying a two-level binomial hierarchical mixed model. They analysed whether income inequality at the state level affects the health of different socio-economic groups differently. Amongst other socio-economic factors, they looked at equivalised household income. They did not find a varying effect of state income inequality on different socio-economic groups, such as e.g. the five income groups they looked at, but an equally negative effect of income inequality on the subjective health of all socio-economic groups. They concluded that there is no association between income inequality and socio-economic health inequalities. On the other hand, in a previous study [20], which examined the subjective health of three income groups (low, middle, and high) in 39 US states, they found a cross-level interaction effect for income inequality and individual income on health.

An analysis of the relationship between wealth and health in 16 countries using the SHARE data is presented in [21]. Across all countries, the authors found a positive and significant effect of wealth on health (net of income), but not for income on health (net of wealth). Health was measured using a Physical Health Index based on 41 items that asked for physical limitations and various illness symptoms. By applying Hierarchical Linear Models, they found a significant cross-level interaction between income inequality and wealth, which supports the thesis that income inequality affects the relationship between wealth and health. However, generalizations should be drawn carefully, since their results were influenced by the special case of the United States. Once the US was excluded from the analyses, the effect of income inequality on the relationship between wealth and health was no longer statistically significant.

### Social policies

Whereas income inequality represents the unequal distribution of earnings, social policies reflect the attempt to redistribute earnings through taxes and social security contributions. These policies aim at increasing individual resources not only through financial benefits but also via social services, e.g., public education, public health, and social security expenditures (social insurance and social assistance). Social policies alleviate the tight budget constraints that impact the lower income strata. Furthermore, people with lower income do not only benefit from targeted social assistance, but also from public investments in general, such as the availability of primary care facilities or public transportation, which are health beneficial [22, 23].

Two possible mechanisms help to determine the relationship between social policies and health inequalities: first, social policies affect health inequalities indirectly by reducing the harmful effects of income inequality on health inequalities; and second, the provision and availability of public services directly promotes health. Health inequalities are reduced, since the lower income group benefits especially from public services. While the first mechanism follows a psychosocial approach, the second follows a neo-material approach.

When applying the various interpretations and operationalisations of welfare state policies, the evidence shows that generous social policies are positively related to population health and reduce health inequalities (for reviews see [2, 3, 24]). Many studies have analysed the relation between the welfare state and population health, mostly by applying a regime approach as a social determinant [2, 3]. In a review article [3], of 73 empirical and comparative studies that discussed the role of politics on population health and health inequalities, the authors specified 31 studies that focused on welfare state generosity as a social determinant. More than half of the evaluated papers in this category found a positive association between welfare state generosity and population health or lower health inequalities. In another review [2], the articles are divided into the Regime approach, which covered welfare state regime typologies; the Institutional approach, which studied policy programs; and the Expenditure approach, which analysed the relationship between social or health spending and average health. Even though the authors could not find results for the Regime approach as consistent as did [3], they found a positive association between generous policies (the Institutional approach) and health in general. Five articles were subsumed under the Institutional approach, which analysed health inequalities. Of these five, only one study found a reduction in income-related health inequalities, while the four other studies did not find that the benefits and social policies had any positive impact on reducing health inequalities. The studies that followed the Expenditure approach found that both social and health spending were associated with smaller socio-economic health inequalities.

In an early review of mostly descriptive studies on socio-economic health inequalities [25], it was found that the Nordic countries—characterised by a Social-Democratic welfare state model that includes higher levels of social benefits and services—did not have lower income-related inequalities in self-rated health. Using data from the European Social Survey 2002 and 2004, another study [26] found that the smallest income-related inequalities in self-rated health occurred not in the Nordic countries, but rather in the Bismarckian countries (e.g., Germany, France, Austria, or Belgium).

The few review studies that exist show that there is little research regarding the impact of welfare institutions or social spending on health inequalities. Only around a third of the studies (9 of the 31) that followed the welfare state approach in the literature review of Muntaner et al. [3] discussed socio-economic inequalities in health. Notably, in the review of Bergqvist et al. [2], half of the reviewed articles (28 of the 54) studied socio-economic health inequalities. Nevertheless, in both articles the authors argued for future research with a focus on the relationship of social spending and the health of different socio-economic groups (see also [27]).

### Hypotheses

When looking at income inequality and its implications for health inequalities, Wilkinson [1] claimed that income inequality negatively affects everyone’s health. This claim seems to suggest that the average population health could worsen, but this reduction would not impact health inequality either positively or negatively. It overlooks the fact that people in the lower income strata are especially vulnerable because they have few buffers to protect them. Income inequality especially impacts the health of the lower income strata, which leads to increasing differences in the health of different socio-economic groups. Thus, I hypothesise that income inequality increases health inequalities, regardless of whether the mechanism is via disintegration or the disinvestment in public services.

Social policies are related to an increase in average population health [3]. In sum, improvements of the health of lower income groups outweigh the redistributive burden for higher income groups (e.g., via higher taxes) [28]. Here, generous social policies are indicated by the extent of social spending. Social spending serves as a proxy for the availability and quality of public welfare. The higher the social spending, the better are public welfare services and institutions [22]. The more that is redistributed from overall economic performance (either in the form of direct benefits or in investments in public spheres, such as education or health care), the greater the benefit for lower income groups. Therefore, I hypothesise that generous social policies reduce health inequalities.

Furthermore, I assume that income inequality and social policies have additive effects on health inequalities, but they do not influence each other’s impact on health inequalities.

## Data and methods

### Data

To test the theoretical expectations outlined so far, I use the European Values Study (EVS) round 4 from 2008 and 2009, which is currently the most complete survey of European countries [29]. The EVS is based on random probability samples with an intended net sample size of 1,500. Depending on the size of the country, the sample size could be lower (e.g., in Iceland and Ireland). The mode of interview is usually face-to-face. For documentation of the data, see [30]. The sample includes 44 countries and is restricted to individuals older than 17. However, due to the non-availability of macro data, particularly the Gini index, Bosnia and Herzegovina and Kosovo are not included in the analyses, which reduced the sample to 42 countries.

Further restrictions arise when individuals have missing values on one of the variables used. I applied list-wise deletion for both the dependent variable and the control variables. For most of the countries, the share of deleted cases ranged between 1 and 10 %. In four countries, the share was between 10 and 15 %. Ireland was an exception with 20 % deleted cases.

In order to study the role of income inequality and social policies on health inequalities I apply a two-step hierarchical estimation, first at the individual level, second at the country level.

### Individual-level variables

The dependent variable for analyses at the first step was subjective general health based on the following question: ‘All in all, how would you describe your state of health these days? Would you say it is… very good, good, fair, poor or very poor?’ Subjective health is a valuable measure for health because it is strongly associated with mortality and functional ability [31–34].

By combining the response categories of subjective health into having very good or good vs. less than good health I follow the approach of other colleagues (see, e.g., [35]). Additionally, to address the problem of losing information when recoding several response categories into a binary variable, I used subjective health with the original 5-point response scale (see also [36, 37]). Since I did not assume an equidistant scale, I considered the 5-point scale of subjective health as an ordinal-scaled variable.

The explanatory variables in the model of the first step are income quartiles. Income was imputed due to the large number of missing values in some countries. The multiple imputation was carried out using the STATA command *mi impute* [38]. Regression equations on household income were run to complete the missing income data based on other available data in the cases. In the linear regression model for the multiple imputation, I included all the variables used in the analyses and an additional auxiliary variable for occupational status using European Socio-economic Classification (ESeC) from the Institute for Social and Economic Research (ISER). I performed a sensitivity analysis by running the models without imputed income values. Besides a slightly higher number of countries showing significant (*p* ≤ 0.05) income-related health inequalities, the results were similar. Household income, counting all types of income after taxes, was originally asked using 12 country-specific answer categories in the EVS [30]. For comparability between countries, the dataset also provided a version of the income variable where it was converted to purchasing power parity (PPP) in Euros. Furthermore, I applied the square root scale to assess equivalised household income. After these adjustments of the income data, income quartiles were calculated.

Further variables which influence health are included as control variables: age (ranges from 17 to 100), sex, living together with a spouse or partner and employment status. With increasing age, probability of poor health, chronic diseases, and constraints in daily activities increase. Sex is also found to be a strong predictor of health. Usually, women report a higher rate of poor health then men. Living together as a couple also may affect health. Not as much as being married, but benefits from the closeness of a life partner makes this variable meaningful to control for. Employment status was represented by a dummy variable for the non-working (retired/pensioned persons, the unemployed, people who are disabled and hence unable to work, and housewives not otherwise employed).

Furthermore, education is an important control variable when studying socio-economic health inequalities, since the effect from income on health might be mediated by this variable. To analyse the independent contribution of income on health, education is adjusted for. Education was measured according to the International Standard Classification of Education (ISCED 97).

### Country-level variables

At the second step, the macro level, the dependent variable is health *inequality*. Health inequality was estimated as the effect of income on subjective health in the first step. Depending on the use of subjective health as a dummy or an ordinal variable, two models were tested.

I used two explanatory variables—income inequality and social policies. Income inequality was measured with the Gini index provided from the Standardized World Income Inequality Database (SWIID) [39]. The SWIID is based on the Luxembourg Income Study (LIS) and offers comparable high quality data [40]. The estimate of Gini index used in this publication is based on equivalised (square root scale) household market income (pre-tax, pre-transfer). Market income was chosen, since the net income includes social transfers, which are measured via the indicator of social policies. Nevertheless, it was found that the choice of indicator for income inequality did not make a difference with respect to determining the relationship between income inequality and mortality [41]. Data is from the respective year in which the surveys were fielded, i.e., from 2008 for most countries, and from 2009 for Belgium, Finland, the UK, Italy, and Sweden.

Several possibilities exist for measuring social policies. For example, a lot of research has used welfare state regime types, which limit the methods of analyses to regime comparisons (see also [3, 26, 42]). Using social spending as an indicator of the generosity of social policies enabled me to apply a quantitative measure that guaranteed at least some comparability.

In order to focus on social spending for people most in need, I used social protection expenditure as percentage of GDP. This indicator consists of ‘transfers, in cash or in kind, by social protection schemes to households and individuals to relieve them of the burden of a defined set of risks or needs’ [43], as well as the administration costs of the management and administration of those specific schemes. Data on social protection expenditures (SPE) was not available from a single source. However, for most countries, data for public social protection expenditures was derived from the European System of integrated Social PROtection Statistics (ESPROSS) from Eurostat [44], and the Social Security Expenditure Database of the International Labour Organization (ILO) [45]. For some countries, data was available from both sources, which enabled me to verify that the numbers, and consequently the underlying concept of the different data sources, were comparable. Comparisons with some national statistics further supported the numbers provided by the ESPROSS database. For some of the Western Balkan countries, data was collected on the basis of publications of the World Bank [46] and the World Health Organization’s (WHO) European Health for All database (HFA-DB) [47]. Also, this data is from 2008 instead of 2007, as it was for the other countries. In the Appendix B I present an overview of the variables and the data source for the numbers of social protection expenditures.

Additionally, I introduced economic performance as a control variable in the models. Economic performance is based on the gross domestic product per capita (GDP p.c.) in purchasing power parities [48]. To reduce the influence of potential outliers, I built averages using data from the years 2007, 2008, and 2009 according to data availability. After confirming the often found curvilinear association of GDP with health [1] with the data in use, I applied the logarithm of GDP p.c.

### Analytical strategy

To measure the influence of the macro determinants on health inequalities, I applied a two-step hierarchical estimation [49–52]. The approach of the two-step hierarchical estimation allows for an analysis of nested data (e.g., individuals in countries) in a straightforward manner. Especially in cross-national opinion research, we can use the fact that each cluster (e.g. countries with over 1000 observations) includes enough observations to allow for a separate analysis [50]. At the first level, variation in the dependent variable is explained by the individual level variables of the specific unit—in this case: the country. At the second level, the first-level parameters (here: the effect of income on health) are implemented as dependent variable in a model also including country-level explanatory variables. For the present study, as a first step, I ran country-wise regressions, both logistic and ordered logistic, since I generated two different basic models: the first model uses as a dependent variable a recoded dummy variable of poor health, and the second uses the original 5-point response categories of subjective health as an ordinal variable. The micro level analyses were weighted by a general weight factor provided in the dataset. The weight adjusts the sample’s characteristics age and sex to their distribution in the national populations [30].

To present the effect of income on subjective health, I used marginal effects at the mean (MEM), since they offer an intuitive interpretation compared to logit coefficients or odds ratios. MEM show how the probability of the occurrence of the dependent variable is predicted to change as the independent variable changes by a unit—holding all other control variables at their means. In the case of the health dummy variable, MEM expresses the difference in the predicted probabilities of ‘less than good’ health as being in the lowest versus the highest income quartile—holding all other variables at their means. The interpretation of MEM for the ordinal dependent health variable (ranging from 1 *very good* to 5 *very poor*) is more complex, since one MEM exists for every response category. To solve this problem, I generated one single indicator, based on the calculation of an index of dissimilarity: for every country, I summed up the absolute value of the five different MEM as being in the lowest versus the highest income group on subjective health. Subsequently, I divided the sum by two. The higher the index, the higher are the health inequalities [53].

In the second step, at the country level, the two indicators of income-related health inequalities, which were estimated in the first step, were used as dependent variables. In the ordinary least squares (OLS) regression models the determinants of health inequalities were introduced one by one. Following this approach, rather than applying simultaneous multilevel analyses, enabled me to consider country specifications and to study outlying cases. Both description and regression diagnostics regarding outlying cases are simplified. The results are presented in standardised regression coefficients. Standardised regression coefficients allow for a comparison of the effects of independent variables with different units of measure.

## Results

### Results from the first step, (ordered) logistic regressions

When running the model using the health dummy ‘less than good health’ as the dependent variable, 23 out of 42 countries displayed significant income-related health inequalities (i.e., the effect on health of being in the lowest income quartile compared to the highest income quartile was significant, p < .05). Countries with non-significant findings were scattered across Europe; there was no cluster found according to specific regions such as, e.g., Scandinavia or Eastern Europe. The highest inequalities are found in Germany: the probability of having less than good health is around 26 percentage points higher for respondents in the lowest compared to the highest income quartile. The lowest significant effect is found in Greece: the probability of having poor health in the lowest income quartile is only seven percentage points higher. The two measures for health inequalities and the significance level of the effect of income on health are found in the Appendix B of the present study.

Applying the country-wise ordered logistic regressions with the original 5-point scale of health showed that 32 countries had significant income-related health inequalities (*p* < .05). The ten countries with non-significant effects of income on health were from all regions of Europe and did not cluster. Denmark stands out with an inverse but not significant effect, i.e., the respondents of the lowest income quartile claimed to have better health than those of the highest income quartile. Similar to the indicator of health inequalities described above, Germany showed, next to Lithuania, the highest health inequalities with an index of dissimilarity (ID) of 22 %. This means that, while holding the control variables at their means, 22 % of the respondents in the lowest income quartile would have to change their response category of health to have a health distribution equal to the highest income quartile. Belgium had the lowest significant health inequalities (an ID of 7 %).

In accordance with previous research, I confirmed the health gradient in income for both the health dummy and the original variable of subjective health for most countries. Not only did the weakest income group assess their health worse than the highest income group, but also the groups in between fell into a similar pattern: the lowest compared to the highest income quartile was the worst off, but the second income quartile was still more disadvantaged than the third quartile is, when compared to the highest quartile.

### Results from the second step

Table 1 presents the results for the first indicator of health inequalities, which was based on the health dummy variable. There is some support for the hypothesis of a positive association (.34) of income inequality and health inequalities: a higher Gini index is related to higher health inequalities, although not at a conventionally significant level (Table 1, Model 1). When the other two macro determinants were introduced (Model 4, 5 and 7), the Gini index gained significance throughout all model specifications.

**Table 1:** Standardised beta coefficients of income-related health inequalities (MEM of ‘less than good health’) on macro determinants, 42 European countries, 2008/09: comparison of macro determinants

	Model 1	Model 2	Model 3	Model 4	Model 5	Model 6	Model 7
Gini index	.336^+^			.354^*^	.425^*^		.425^*^
	(.056)			(.038)	(.011)		(.013)
Social Protection		-.248		-.267^+^		-.0923	-.0244
Expenditures in %		(.113)		(.079)		(.652)	(.900)
GDP, logged			-.297^+^		-.390^*^	-.236	-.374^+^
			(.056)		(.011)	(.251)	(.063)
Number of cases	42	42	42	42	42	42	42
R^2^	.119	.0616	.0883	.190	.263	.0931	.265
adjusted R^2^	.097	.0381	.0655	.149	.226	.0466	.207

Standardised beta coefficients; *p*-values in parentheses

^+^ *p* < 0.10, ^*^ *p* < 0.05, ^**^ *p* < 0.01, ^***^ *p* < 0.001 (two-tailed tests)

Source (dependent variable): EVS (round 4) [29]; data weighted using a sampling weight. Income-related health inequalities adjusted for age, sex, living together, education, and employment status

(independent variables): IMF [48], SWIID [39], EUROSTAT [44], ILO [45], ADB [65], WHO [47], WB [46]

The relation between social protection expenditures and health inequalities could not be confirmed. As expected, SPE and health inequalities are negatively related (−.25), but the relation does not reach significance (Table 1, Model 2). When running the analysis in which both GDP p.c. (logged) and SPE were introduced as macro determinants (Model 6), the standardised regression coefficient of SPE on health inequalities was heavily reduced, which indicated an importance of GDP over SPE.

GDP p.c. (logged) had a negative effect on health inequalities meaning that economic performance reduced income-related health inequalities (Table 1, Model 3). The effect of economic performance on health inequalities was linked to income inequality (Model 5). While controlling for the Gini index, the standardised regression coefficient of GDP p.c. (logged) on health inequalities increased and was significant at a higher level. Still, income inequality was the dominating explanatory effect for health inequalities: models including the Gini index showed the highest adjusted R^2^. Furthermore, the Gini index appeared as the highest standardised regression coefficient compared to the log of GDP p.c. and SPE.

**Fig. 2:**
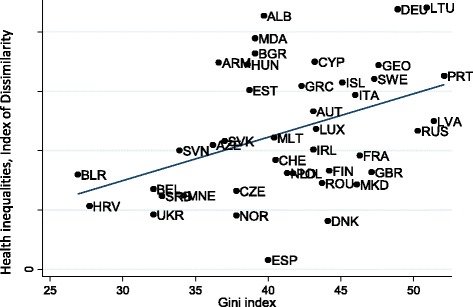
Scatter plot of health inequalities against the Gini index in 42 European countries. Health inequalities are measured using the index of dissimilarity. Linear regression equation and coefficients are *y* = −0.016 + 0.003*β, r = .39 (p* < 0.05)

When income-related health inequalities were analysed based on the index of dissimilarity as the dependent variable in the model, the results were similar (Table 2). Running models with each macro determinant separately, the direction and size of the coefficients were found to be very similar to those in the models discussed above. The Gini index was positively related to health inequalities (.39, p < .05), i.e., higher income inequality was linked to higher health inequalities (Table 2, Model 1). Fig. 2 illustrates the relation between health inequalities and income inequalities in 42 European countries.

**Table 2:** Standardised beta coefficients of income-related health inequalities (Index of Dissimilarity) of macro determinants, 42 European countries, 2008/09: comparison of macro determinants

	Model 1	Model 2	Model 3	Model 4	Model 5	Model 6	Model 7
Gini index	.390^*^			.409^*^	.464^**^		.455^**^
	(.022)			(.013)	(.006)		(.008)
Social Protection		-.252		-.274+		-.190	-.117
Expenditures in %		(.107)		(.066)		(.360)	(.547)
GDP, logged			-.219		-.320^*^	-.0933	-.241
			(.163)		(.034)	(.652)	(.225)
Number of cases	42	42	42	42	42	42	42
R^2^	.158	.0635	.0480	.233	.255	.0685	.264
adjusted R^2^	.137	.0401	.0242	.194	.217	.0207	.206

Standardised beta coefficients; *p*-values in parentheses

^+^ *p* < 0.10, ^*^ *p* < 0.05, ^**^ *p* < 0.01, ^***^ *p* < 0.001 (two-tailed tests)

Source (dependent variable): EVS (round 4) [29]; data weighted using a sampling weight. Income-related health inequalities adjusted for age, sex, living together, education, and employment status

(independent variables): IMF [48], SWIID [39], EUROSTAT [44], ILO [45], ADB [65], WHO [47], WB [46]

Also, higher social protection expenditures were related to reduced health inequalities (−.25, n.s.) (Table 2, Model 2). The non-significant findings between SPE and health inequalities are not due to the small number of cases, namely 42 countries, as the correlation between SPE and average population health was significant (p < .001). When compared to the other indicator of health inequalities discussed above, GDP p.c. (logged) was not significantly correlated with health inequalities this time (Table 2, Model 3). Again, income inequality was the dominating explanatory effect for health inequalities.

### Sensitivity Analysis

When a sample is small, such as 42 countries in this case, single data points could be critical for estimating the regression and interpreting the effects of the results [54]. When operationalising health inequalities, using the dummy variable of poor health, the case of Germany stands out, since it has the highest income-related health inequalities, but ranges in the middle of the three macro determinants. Previously, lower or medium health inequalities were found in Germany [26, 55]. Regression diagnostics showed a noticeable overall influence (Cook’s Distance, DFFITS) of the data point of Germany due to large residuals. Estimating the analyses without the exceptional case of Germany gives different, i.e., more significant, results: the effect of social protection expenditures on health inequalities increases and becomes significant at the 5 % level. Similar modifications can be found for the effect of GDP (logged) on health inequalities. The Gini index is not significant.

Nevertheless, the analysis of 41 countries, excluding the outlying case of Germany, supports the conclusions of the previous analyses. Even though income inequality as a single determinant no longer plays a significant role in health inequalities, the pattern is again very similar to the analyses of all 42 countries. However, it is notable that GDP (logged) seems to be the most important determinant for the association to income-related health inequalities, contrary to the findings based on all 42 countries.

Implementing the index of dissimilarity as the dependent variable, regression diagnostics showed that Moldova might have an overall influence on the regressions, but a regression analyses that excluded the case of Moldova did not offer any different insights than running the analyses with all 42 countries.

## Discussion

The first hypothesis was that income inequality increases health inequalities. In all models and with both versions of the health inequality indicators this could be confirmed. The Gini index appeared as the only independent variable showing a stable significant relation with health inequalities throughout all model specifications.

The second hypothesis regarding social policies is not confirmed. Social protection expenditure is not significantly related to health inequalities even though the coefficients are in the expected direction: Higher social protection expenditures are related with lower health inequalities. Since social protection expenditures are correlated with average population health (.49, p < .001, own analysis) it appears that social policies have a health-promoting impact for all of society—though not specifically for certain groups in need, e.g. the lower income groups. Social policies contribute to better population health but do not show a reducing effect on health inequalities.

As a third hypothesis, I assumed that income inequality and social policies have additive effects on health inequalities. This assumption can neither be declined nor confirmed, since both income inequality and social protection expenditures do influence each other’s impact on health inequalities only slightly (Model 4 compared to Model 1 and 2, respectively). On the one hand, this speaks against the psychosocial mechanism of the relation between social policies and health inequalities. Harmful effects of income inequality on health inequalities are only slightly balanced by social policies (Model 4). On the other hand, the neo-material mechanism, i.e. that the availability of public services directly reduces health inequalities because lower income groups benefit the most, seems to play a part, as economic performance reduces the impact of social policies on health inequalities (Model 6).

Regarding the control variable ‘economic performance’, the findings show a negative link between GDP p.c. (logged) and health inequalities, which means that higher economic performance is related to lower health inequalities. This is contrary to previous studies that found only weak or no associations between GDP p.c. (logged) and health inequalities [11, 21]. However, a specific of this study is the EVS data which comprises a wide range of countries with various levels of national income (see Appendix B). Some countries are indeed at a lower stage of economic development, where additional GDP matters for the reduction of health inequalities—contrary to the country selections of the above mentioned studies.

In the introduction, I described two processes of distribution of national income. The analyses show that only the distribution of personal earnings, measured by the Gini index, seems to play a role regarding health inequalities. Redistribution via social policies, measured by social protection expenditures, does not reduce health inequalities. Consequently, when thinking about reducing income inequality in order to reduce health inequalities, social policies do not seem to be the best fit to balance out unequal incomes. However, the reason is the mechanism of how social policies affect health inequalities rather than the mechanism of redistribution by itself. According to Dallinger [56], government income redistribution works effectively in the way that indeed the lowest income group benefits from public redistribution while the highest income group experiences income losses. The middle class holds its position. Even though social policies are targeted towards lower income groups, they might be too diverse in their impacts to show a distinct health-promoting benefit for disadvantaged income groups. However, to solve this question, further research on specifically health-promoting effects of various social policies is necessary.

### Strengths and limitations

With respect to future studies, the limitations of this study should be discussed. In 2008, the European Values Study covered the whole geographical area of Europe. Although the EVS represents a unique dataset that integrates various European societies, it may include field work that varies in quality across different countries.

For macro-comparative analyses, low numbers of units of analysis are typical [3]. In this case, the number of countries analysed (42) was an inevitable constraint that should be kept in mind when interpreting the results. Usually, to study people nested in countries, the typical approach is to use simultaneous multilevel analysis; instead, to gain more detailed information on single countries, I used a two-step approach—I extracted country-specific effects of household income on subjective health from the micro level at the first step, and subsequently introduced them as dependent variable at the macro level in the second step. This led to the finding of the outlying case of Germany: high health inequalities are combined with a medium level of Gini index, social expenditures, and GDP, as well as medium subjective health at the mean (see Appendix A and Appendix B). Future research could show if this is a specific finding and hence an artefact of the EVS data, or whether income-related health inequalities did indeed increase compared to findings based on earlier data.

Since little research has used a comparative approach to focus on *inequalities in* health [51], an agreement on the best indicator for socio-economic health inequalities does not yet exist. Subjective health includes both the physical and mental aspects of health. Even though it is often criticised because it is based on individual perceptions, subjective health is widely used in research on population health as well as health inequalities [57]. Since this present study is based on within-country income-related health inequalities, cross-national differences in response styles of self-assessed health [58] are negligible. The question whether socio-economic factors such as income influence respondents’ self-assessment of health, which would bias the estimation of health inequalities, is not solved yet. Jürges [59] finds that response behaviour varies according to socio-economic groups. On the other hand, Van Doorslaer and Gerdtham [60] conclude that income-related health inequalities are ‘unlikely to be biased by such reporting tendencies’ (p. 14).

A strong point of this present study is that it tests two different dependent health variables in country-specific models at the first step. When using the effect of income on health, both health variables have certain advantages and disadvantages as indicators of health inequalities. The interpretation of marginal effects at the mean is more straightforward when running regressions on the health dummy variable. However, after combining the categories, less information was obtained compared to using the original 5-point response scale; also, the way the categories were combined is perhaps controversial. Therefore, health inequalities were also calculated on the basis of a dummy variable of (very) poor health versus fair and (very) good health as recommended by Etilé and Milcent [61]. Probably due to the rather low share of respondents with (very) poor health, only a few countries displayed significant income-related health inequalities. Since it was questionable as to whether this health dummy was an appropriate indicator for health inequalities if it targeted such a small number of respondents, I decided against presenting those results.

Regarding the index of dissimilarity as an indicator of health inequalities, I discovered that using the original 5-point response scale as a metric rather than an ordinal variable led to approximately equivalent results at both the first and second step.

Studying income-related health inequalities across countries imposes the challenge to generate one variable for income across a variety of countries. In this case, the variable had to ensure that respondents’ income in Luxembourg was comparable to respondents’ income in Moldova—to name two extreme cases. Additionally, some countries had a high rate of missing values. Both factors were taken into account when computing the income variable but nevertheless could be interpreted as a limitation of this study. For future studies, education instead of income might be an interesting measure for socio-economic health inequalities. However, given that half of the EVS dataset consists of post-communist countries, where a good part of the adult population was educated during Communism and equal access to education was emphasised [62], educational health inequalities would need to be interpreted carefully, for they might not adequately describe socio-economic inequalities.

While the Gini index is a widely used and recognised indicator for income inequality, one single predominant measure for the impact of social policies in comparative health inequality research is missing. Dahl and van der Wel ([63], p. 60) even claimed that ‘a social expenditure approach is new in this field of research.’ Using social protection expenditures in the percentage of GDP as a quantitative measure for social policies should be understood as just a starting point for further analyses. The number of various countries in the EVS made it impossible to find one single data source for social protection expenditures. However, with Eurostat, I found a database encompassing 30 countries (see Appendix B). Furthermore, I took reasonable care in data investigation for the other countries and tried to double-check with other sources, e.g., national statistics. Although social protection expenditures already are a specification of the comprehensive understanding of social policies, it would be interesting for future research to look at the effects of schemes of social protection, i.e., minimum income protection, on health inequalities.

## Conclusion

The present study investigates the importance of macro determinants for reducing income-related health inequalities. In particular, the aim of the study is to analyse the role of income inequality and social policies as determinants of health inequalities. As found in earlier studies [64], the Gini index plays an important part when studying the relations between the macro determinants and health inequalities. When comparing the standardised regression coefficients, the Gini index has the largest effect throughout all model specifications, even though interpretations of non-significant effects have to be considered carefully. Income inequality has more impact on health inequalities than social protection expenditures, independent of the design of the health variable used as the base for health inequalities. Even though the findings were not as clear as desirable, due to non-significance, the results show the negative association between social policies and health inequalities as expected. Overall, the power of redistribution within societies to impact income-related health inequalities should not be downplayed, yet increases in national income do not automatically lead to reduced health inequalities. The redistribution of income and economic resources plays part in reducing health inequalities, as it depends on the extent to which the population benefits from increased GDP through redistribution.

## Appendix A

**Table 3:** Sample sizes and individual-level variables

	EVS sample size^a^	Age mean	Age Std. Dev.	Sex proportion of male respondents	Subjective health mean
Albania	1,388	41.1	14.8	0.49	3.74
Armenia	1,424	44.0	17.6	0.43	3.38
Austria	1,485	46.5	17.6	0.43	3.98
Azerbaijan	1,450	34.4	12.4	0.50	3.67
Belarus	1,435	42.3	17.1	0.40	3.27
Belgium	1,498	48.1	17.4	0.48	3.96
Bulgaria	1,406	50.2	17.6	0.43	3.49
Croatia	1,319	45.4	18.2	0.40	3.60
Cyprus	993	49.9	18.6	0.44	4.00
Czech Republic	1,693	48.5	18.2	0.46	3.73
Denmark	1,422	50.0	16.6	0.50	4.21
Estonia	1,510	50.2	18.5	0.35	3.44
Finland	1,049	47.3	14.9	0.48	3.63
France	1,491	50.0	18.3	0.46	3.87
Georgia	1,482	45.6	17.1	0.37	3.33
Germany	1,877	49.7	16.5	0.47	3.71
Great Britain	1,457	51.5	18.9	0.42	3.91
Greece	1,451	49.6	18.5	0.43	4.03
Hungary	1,466	44.6	17.7	0.48	3.49
Iceland	690	44.7	16.1	0.51	4.16
Ireland	813	45.9	17.6	0.41	4.31
Italy	1,392	47.6	18.0	0.49	3.81
Latvia	1,407	46.9	18.4	0.37	3.33
Lithuania	1,433	46.6	17.9	0.45	3.41
Luxembourg	1,565	39.7	17.5	0.49	4.09
Macedonia	1,307	44.3	15.9	0.57	3.96
Malta	1,468	52.2	17.8	0.37	3.76
Moldova	1,490	45.1	17.6	0.46	3.17
Montenegro	1,418	42.6	16.4	0.45	3.69
Netherlands	1,494	54.8	17.4	0.45	3.92
Norway	1,081	45.7	16.1	0.51	4.10
Poland	1,384	44.5	17.0	0.45	3.66
Portugal	1,490	52.6	18.7	0.40	3.48
Romania	1,323	48.1	17.1	0.44	3.50
Russia	1,427	46.2	17.8	0.33	3.09
Serbia	1,364	45.9	16.8	0.47	3.53
Slovak Republic	1,443	53.6	16.5	0.40	3.36
Slovenia	1,291	48.8	17.9	0.46	3.64
Spain	1,456	47.8	19.2	0.44	3.93
Sweden	1,011	49.4	14.7	0.48	4.00
Switzerland	1,227	49.8	17.8	0.46	4.08
Ukraine	1,486	47.9	17.8	0.38	3.10

^a^After list-wise deletion of the dependent and control variables and multiple imputations on household income. The share of deleted cases ranges between 1 % and 10 %. Ireland with 20 % deleted cases is an exception

Sources: EVS (round 4) [29]

### Appendix B

**Table 4:** Country-level variables

	Health inequalities				GDP (PPP) p.c.	Gini index	Social protection expenditure in % of GDP	Sources of SPE data
	MEM of 'less than good' health		Index of dissimilarity					
Albania	0.233	***	0.213	***	6,799	39.7	10.4	ILO
Armenia	0.194	***	0.174	***	5,373	36.6	4.2	ADB
Austria	0.141	***	0.133	***	38,941	43.1	27.8	EUROSTAT
Azerbaijan	0.084	+	0.105	**	8,687	36.2	3.3	ADB
Belarus	0.089	*	0.080	*	12,091	26.9	18.1	ILO
Belgium	0.075	+	0.068	*	35,692	32.1	26.9	EUROSTAT
Bulgaria	0.195	***	0.182	***	12,650	39.1	14.1	EUROSTAT
Croatia	0.046	n.s.	0.053	n.s.	18,032	27.7	21.7	ILO
Cyprus	0.152	**	0.175	***	28,445	43.2	18.2	EUROSTAT
Czech Republic	0.060	n.s.	0.066	+	24,545	37.8	18.0	EUROSTAT
Denmark	-0.002	n.s.	0.041	n.s.	36,695	44.1	30.7	EUROSTAT
Estonia	0.162	**	0.151	**	19,661	38.7	12.1	EUROSTAT
Finland	0.108	+	0.083	n.s.	34,955	44.2	25.4	EUROSTAT
France	0.128	***	0.096	**	33,735	46.3	30.9	EUROSTAT
Georgia	0.227	***	0.172	***	4,786	47.6	6.4	ILO
Germany	0.264	***	0.219	***	34,890	48.9	27.8	EUROSTAT
Great Britain	0.068	n.s.	0.082	*	35,345	47.1	24.7	EUROSTAT
Greece	0.068	*	0.154	***	29,552	42.3	24.8	EUROSTAT
Hungary	0.162	**	0.172	***	18,841	38.6	22.7	EUROSTAT
Iceland	0.084	n.s.	0.157	**	39,504	45.1	21.4	EUROSTAT
Ireland	0.032	n.s.	0.101	n.s.	41,254	43.1	18.3	EUROSTAT
Italy	0.161	**	0.147	***	29,902	46.0	26.6	EUROSTAT
Latvia	0.113	*	0.125	**	16,321	51.4	11.3	EUROSTAT
Lithuania	0.249	***	0.220	***	17,939	50.9	14.4	EUROSTAT
Luxembourg	0.116	**	0.118	**	81,179	43.3	19.3	EUROSTAT
Macedonia	0.048	n.s.	0.071	*	9,383	46.1	14.1	ILO
Malta	0.100	*	0.111	***	23,930	40.4	17.7	EUROSTAT
Moldova	0.194	***	0.195	***	2,859	39.1	17.5	ILO
Montenegro	0.033	n.s.	0.062	n.s.	10,572	34.2	17.6	WB
Netherlands	0.091	**	0.081	*	40,343	41.3	28.3	EUROSTAT
Norway	0.056	n.s.	0.045	n.s.	52,308	37.8	22.5	EUROSTAT
Poland	0.076	n.s.	0.081	*	17,347	41.7	18.1	EUROSTAT
Portugal	0.186	***	0.163	**	22,812	52.1	23.9	EUROSTAT
Romania	0.070	n.s.	0.073	n.s.	12,012	43.7	13.6	EUROSTAT
Russia	0.114	*	0.116	***	15,293	50.3	12.1	ILO
Serbia	0.097	+	0.062	n.s.	10,463	32.7	22.9	WB/WHO
Slovak Republic	0.070	n.s.	0.108	*	21,162	37.0	16.1	EUROSTAT
Slovenia	0.084	n.s.	0.100	*	28,397	33.9	21.3	EUROSTAT
Spain	0.041	n.s.	0.008	n.s.	30,323	40.0	20.8	EUROSTAT
Sweden	0.117	**	0.160	***	37,039	47.3	29.2	EUROSTAT
Switzerland	0.101	**	0.092	*	40,721	40.5	25.1	EUROSTAT
Ukraine	0.054	n.s.	0.046	n.s.	6,903	32.1	22.7	ILO

*n.s*. not significant, + *p* < 0.10, **p* < 0.05, ***p* < 0.01, ****p* < 0.001 (two-tailed tests)

Sources: EVS (round 4) [29], IMF [48], SWIID [39], EUROSTAT [44], ILO [45], ADB [54], WHO [47], WB [46]

## Abbreviations

ADB: Asian Development Bank
ESeC: European Socio-economic Classification
ESPROSS: European System of integrated Social PROtection Statistics
EVS: European Values Study
GDP p.c.: Gross domestic product per capita
HFA-DB: European Health for All database
ID: index of dissimilarity
ILO: International Labour Organization
IMF: International Monetary Fund
ISCED 97: International Standard Classification of Education, revision in 1997
ISER: Institute for Social and Economic Research
LE: life expectancy
LIS: Luxembourg Income Study
MEM: marginal effects at the mean
OLS: ordinary least squares
PPP: purchasing power parity
SES: socio-economic status
SPE: social protection expenditures
SWIID: standardized world income inequality database
WB: World Bank
WHO: World Health Organization
